# Mixed reality with 3D brain imaging for patient consultation in neurosurgery: an IDEAL stage 2a feasibility study

**DOI:** 10.1007/s11060-025-05047-4

**Published:** 2025-04-28

**Authors:** Vassili Crispi, Samuel Peat, William S. Bolton, Stephen Chapman, Nikki Rousseau, Faisal Mushtaq, Ryan K. Mathew

**Affiliations:** 1 Department of Neurosurgery, Leeds General Infirmary, Leeds, UK; 2 School of Medicine, University of Leeds, Leeds, UK; 3 Centre for Immersive Technologies, University of Leeds, Leeds, UK; 4 Leeds Institute for Medical Research, University of Leeds, Leeds, UK; 5 School of Psychology, University of Leeds, Leeds, UK; 6 Leeds NIHR Biomedical Research Centre, Leeds, UK

**Keywords:** Neurosurgery, Mixed reality, Immersive technology, Consultation, Informed consent

## Abstract

**Background:**

This study aimed to investigate the feasibility of introducing additional Mixed Reality (MR) visualisation of patient-specific imaging during the neurosurgical consultation to improve patients’ understanding, as well as the potential benefits from a patient’s perspective to the consultation process.

**Methods:**

An IDEAL Stage 2a feasibility randomised controlled trial was conducted involving patients with radiologically suspected brain tumours at a large, tertiary UK neurosurgery institution. Patients were randomised into two groups: standard 2D monitor versus additional MR visualisation. Primary feasibility outcomes included recruitment rates, MR intervention adherence, fidelity, and acceptability. Secondary outcomes included patient-reported experiences. Quantitative and qualitative analyses were performed via validated questionnaires.

**Results:**

A total of 36 patients were randomised over 12 months, 17 to a Mixed Reality (MR) Group and 19 to a standard 2D monitor-only group, with no significant baseline differences. MR intervention was deemed feasible for further clinical evaluation with high fidelity and user acceptability. Patients in the MR group reported statistically higher satisfaction with information received, an improved patient-doctor relationship, greater confidence in decision-making, and a better understanding of their condition compared to the standard 2D monitor-only group. No major technological issues were encountered. No adverse effects were reported or observed (including cybersickness). Patients found the MR technology easy to use.

**Conclusions:**

Our findings suggest that incorporating MR visualisation into routine neurosurgical consultations is feasible and offers potential benefits for patients. With minor modifications to the intervention and assessments, we aim to perform a larger-scale, multi-centre randomised feasibility trial, which will also address implementation challenges for widespread adoption and provide more indication of efficacy.

**Supplementary Information:**

The online version contains supplementary material available at 10.1007/s11060-025-05047-4.

## Background

Informative consultations and consent are fundamental ethical principles in clinical practice and essential for good patient-centred care [1, 2]. Enhancing patients’ understanding of their illness leads to greater autonomy, health empowerment, better-informed consent, better communication, and increased trust in the patient-doctor relationship [3–5]. Additionally, these factors maximise post-operative recovery and achieve better outcomes [5].

In neurosurgery, complex neuroanatomy, pathologies, and intricate procedures demand sophisticated imaging techniques, such as magnetic resonance imaging (MRI), which are not translated into comprehensible information for patients [6, 7]. Even for medical students and doctors, neuroanatomy and imaging are challenging, requiring an individual to mentally appreciate 3-dimensional anatomy from the 2-dimensional imaging visualisation [8, 9]. Whilst attempting to understand neuroanatomy, pathologies and proposed surgical procedures, patients retain only 20–60% of information provided [10, 11]. This is also impacted by age and levels of anxiety, especially in neuro-oncology due to the significance and subsequent anxiety associated with a brain tumour diagnosis [11, 12]. This brings into question patients’ understanding of their diagnosis, potentially impacting decisions made following a neurosurgical consultation.

Interactive interventions, including patient-specific 3D printed models, videos, and stereoscopic visualisation, have been shown to increase patient understanding and improve confidence, especially for consent [13–16]. Similarly, immersive technology can enable better 3D visualisation of anatomy. For example, virtual reality (VR) has been successfully evaluated in gaining consent for neurosurgical patients, and mixed reality (MR) has demonstrated promising results for multidisciplinary planning of awake craniotomy surgery [14, 17, 18]. Furthermore, MR was demonstrated to enhance the spatial understanding of brain tumours compared to MRI and 3D models on a monitor in a group of students and neurosurgeons [19].

Nonetheless, VR and MR adopt different technologies: whilst VR projects the user into a virtual world, MR projects virtual objects into the user’s surrounding environment providing a reference point, a process which is called anchoring. Thanks to anchoring, MR has reduced incidences of cybersickness, and nausea compared to VR [17, 20, 21]. Additionally, Brainlab AG (Munich, Germany) has partnered with MagicLeap (MagicLeap Inc, Delaware) to use their commercially available MR headsets for 3D visualisation, leveraging the Brainlab neuronavigation systems already installed in neurosurgical units worldwide. However, there is no evidence for MR use to enhance patients’ understanding in neurosurgery.

This study aimed to investigate the feasibility of introducing additional MR visualisation of patient-specific imaging during the neurosurgical consultation and the potential benefits from a patient’s perspective to the consultation process.

## Methods

This is a prospective, single-centre, open-label, feasibility, randomised controlled trial (IDEAL Stage 2a). Ethics approval was provided by the Health Research Authority (22/NW/0061) and Leeds Teaching Hospital NHS Trust Ethics Committee (IRB approval n. 295487). No changes to the study protocol were made after the trial had started. The study is reported in line with CONSORT guidelines [22].

### Study team

One Academic Consultant Neurosurgeon, author RKM, specialising in Neurosurgical Oncology was involved in both arms of the trial to minimise variation in clinical practice and interpersonal communication. Authors VC and SP were responsible for randomisation, imaging processing and visualisation, and MR hardware use.

### Study design

**Fig. 1:**
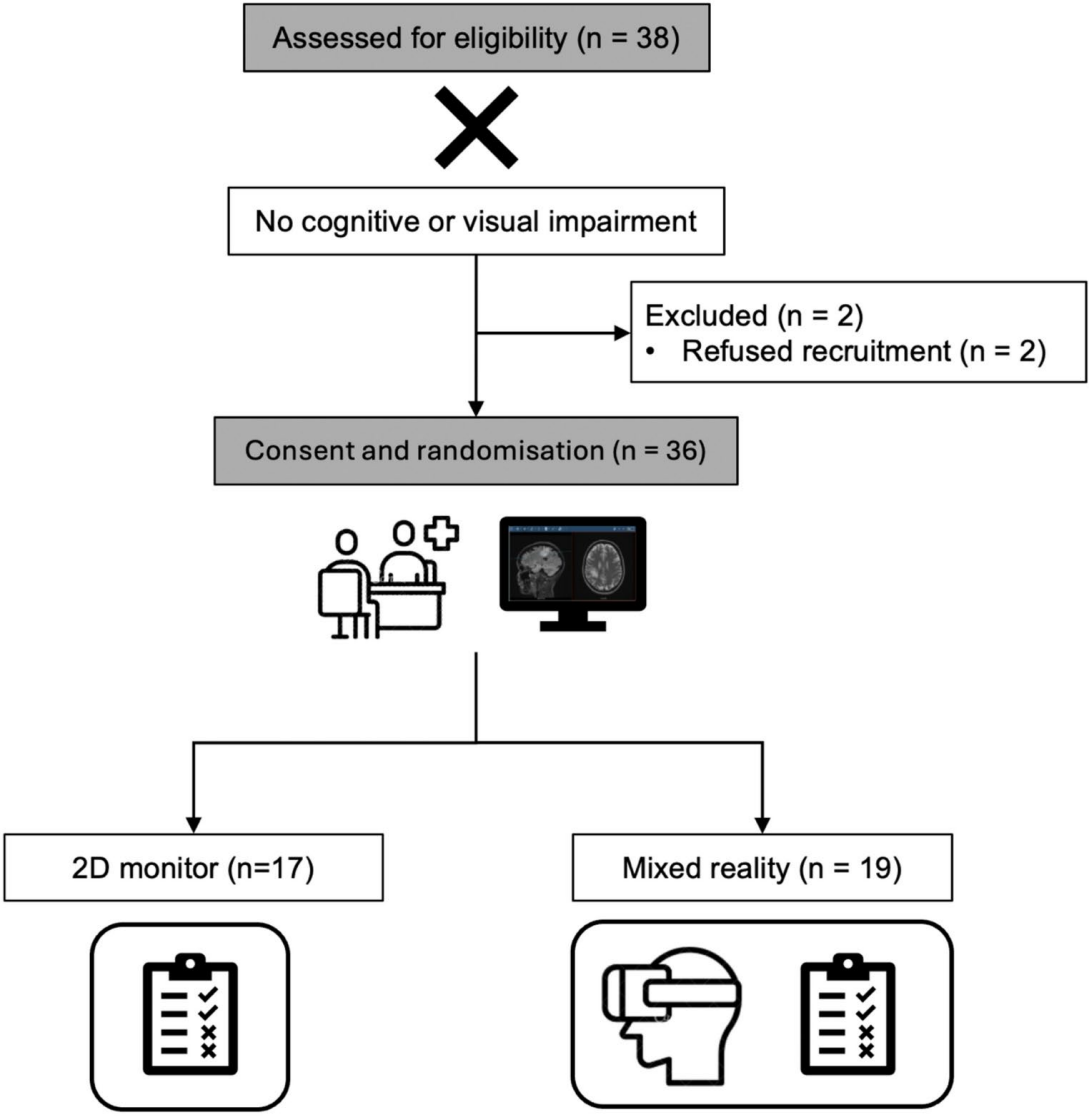
Consort trial participant flow diagram of study design and patient inclusion. The 2D group received the surgical informed consent in a conventional manner utilising the pre-operative 2D Magnetic Resonance Imaging (MRI) sequences, while the MR group received an additional mixed reality visualisation with Magic Leap before consent

All patients referred to a single tertiary neurosurgical institution with new radiologically diagnosed tumours were screened for eligibility. Exclusion criteria were cognitive or visual impairment other than the use of glasses or contact lenses. Patients were recruited from February 2023 to February 2024 (Fig. 1).

All patients underwent a standard consultation, review of pre-operative greyscale MRI imaging on a 2D monitor, and discussion of management options. If applicable, all patients gave their surgical informed consent to author RKM after being informed about the procedure in a conventional way, as described, prior to the randomisation. Eligible patients were randomised by authors VC or SP using a random number generator in Microsoft Excel 2023 (Microsoft, Redmond, WA) to 2D Group (without further intervention) or MR Group (additional holographic MR visualisation of their imaging and explanation of their pathology).

### Image processing

**Fig. 2:**
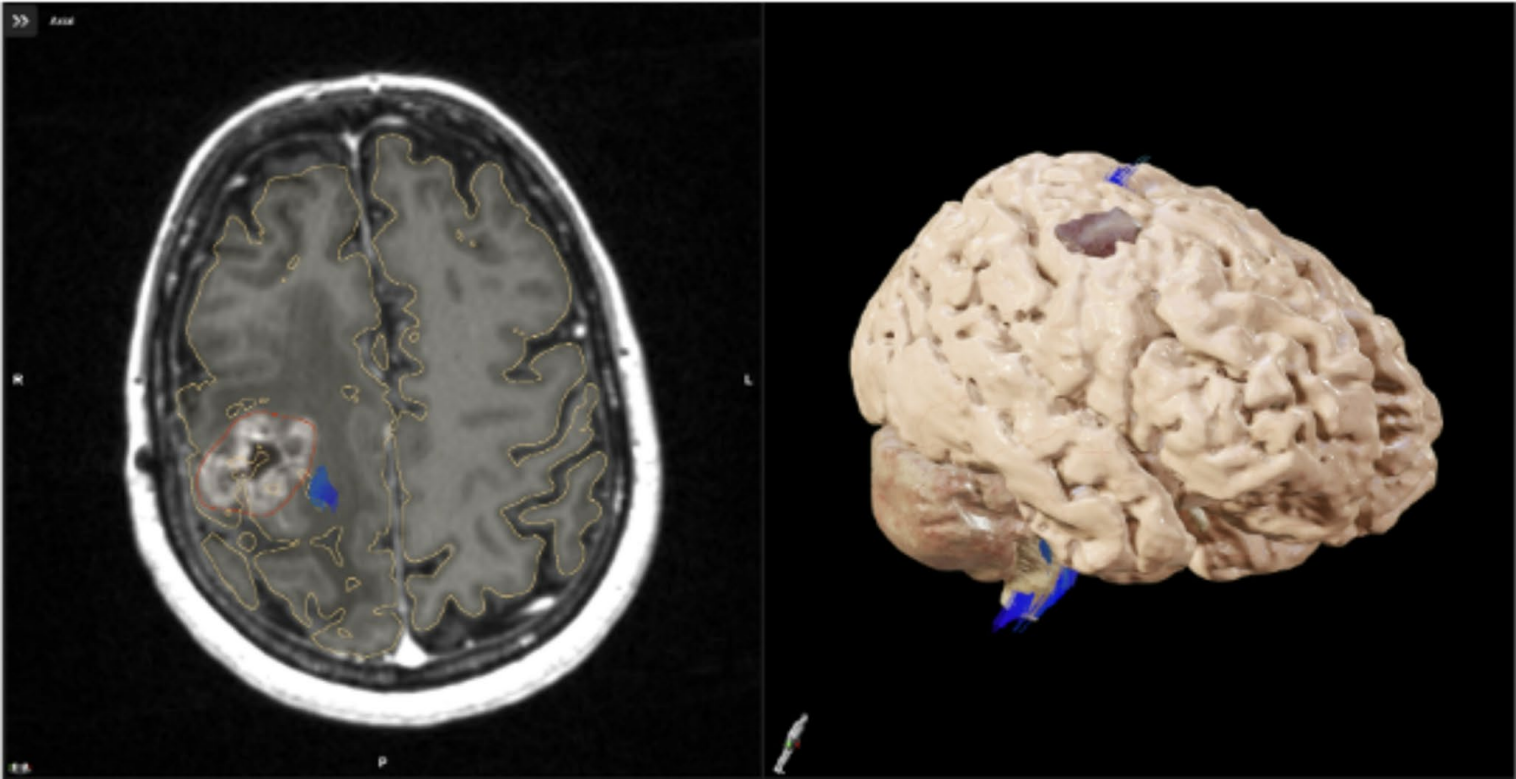
Screenshots demonstrating the planning sequences for the MagicLeap mixed reality visualisation using the BrainLab system. The screenshots (left: Magnetic Resonance Imaging (MRI) with auto-segmentation of cerebral volumes, tumour volume, and fibre tract bundles; right: 3D reconstruction resulting from A). Demonstration video available from these sequences (Supplementary materials)

Brainlab Elements v3.3.1 cranial planning workflow was used to generate a volumetric reconstruction of the intracranial anatomy via auto-segmentation of pre-operative MRI sequences (Fig. 2). Adopted MRI sequences were available as part of routine clinical care as no additional imaging was performed: T1 with contrast enhancement (CE) and Fluid Attenuated Inversion Recovery (FLAIR) (*n* = 12), T1 and T1 with CE (*n* = 1), T1 and FLAIR with CE (*n* = 2), T1 and FLAIR with fat suppression (*n* = 1), T1 and FLAIR (*n* = 2), T1 with CE and T2 (*n* = 1). MRI sequences with a minimum of 150 slices were adopted to increase auto-segmentation accuracy.

Automated superimposition of selected MRI sequences with proprietary atlas-based anatomical landmarks was performed. The operator was then required to manually highlight the tumour in the axial, coronal and sagittal planes. The cerebrum, cerebellum, brainstem, ventricles, and optic chiasm were automatically segmented by the software. All sequences were visually checked by authors VC or SP. No adjustments were required.

A single training session was required by the study team prior to using the Brainlab software and the MR hardware.

### Interventions

MagicLeap headsets were set in standby mode during the early stages of the consultation. Once required for use in the MR Group, the headsets were sequentially re-activated and synced with the patient-specific sequences via a QR code using Brainlab Elements v3.3.1. These sequences were visualised through concurrently worn MR hardware by author RKM and the patient. Both surgeon and patient viewed the same, synchronised 3D holographic reconstruction in the clinic room, and author RKM explained the pathology and/or proposed surgery in accordance with routine clinical practice (Supplementary material, Video 1).

### Outcomes

The primary outcomes were the feasibility of introducing additional MR visualisation of patient-specific imaging during the neurosurgical consultation to inform future studies. Feasibility outcomes included recruitment, intervention fidelity, and data collection instrument completion rate. Recruitment success was defined as 80% of eligible patients agreeing to be enrolled. Successful intervention fidelity was defined as at least 80% of the MR group completing the consultation, for example completing the MR visualisation of patient-specific sequences. Successful instrument completion rate was defined by at least 80% of data collection tools being completed. Successful retention was defined by less than a 10% attrition rate. Methodological issues in this feasibility study were assessed using the 14 common methodological issues in feasibility studies identified by Shanyinde et al. [23, 24].

Secondary outcomes evaluated any potential benefits from a patient’s perspective to the consultation process, including benefit of consultation type (2D versus MR) perceived by the patient on (1) information received and decision-making; (2) their understanding of their condition; (3) patient-doctor relationship during the consent process (if appropriate, as not all consultations included consent).

### Outcome instruments and data collection

After the conclusion of the clinic, both groups were requested to complete a questionnaire. Section A was completed by authors VC or SP and included demographic details, e.g. age, gender, handedness, education level, previous use of immersive technology, tumour type, and tumour location. Additionally, duration of use of MR technology and incidence of any technological issues encountered were collected.

Following sections consisted of standardised statements, and patients were asked to state their agreement from 1 (strongly disagree) to 5 (strongly agree): (B) 5-point Likert-type, 10-item COMRADE scale (Combined Outcome Measure for Risk communication And treatment Decision-making Effectiveness), and (C) 5-point Likert-type, 20-item educational evaluation scale [25]. The MR group answered additional questions on (D) 16-item modified Simulator Sickness Questionnaire and 5-point Likert-type, modified 8-item System Usability Scale (m-SUS) [26, 27].

The COMRADE scale explored patients’ self-reported satisfaction with communication, patient-doctor relationship, and confidence in the decision reached, and the m-SUS reported patients’ perception of MR hardware complexity, ease of use, and level of confidence in using the device. An adapted questionnaire on simulator sickness explored patients’ exposure to potential symptoms associated with the use of immersive technologies as none, slight, moderate, or severe [26].

At the end of the questionnaire, patients were offered to provide open feedback: written feedback was completed by the patient, and verbal feedback was transcribed by authors VC or SP.

### Sample size

As a feasibility study, a power calculation was not performed. Based on available literature, a target sample population of 36 patients was determined to obtain sufficient data to assess feasibility outcomes [28].

### Data analysis

Statistical analysis was performed using SPSS v29.0 (IBM Corp., Armonk, NY). Normality of data distribution was ascertained using the Shapiro-Wilk test. Mean (± SD) or median (IQR) were calculated for parametric and non-parametric data, respectively. Statistical analysis was undertaken with independent t-test, Mann-Whitney U test and Pearson Chi-Square test. Graphs were created using GraphPad Prism v10.1 (Dotmatics, Boston, MA). Statistical significance was determined as *p* < 0.05.

Qualitative analysis of the verbal and written feedback was undertaken using content analysis to identify recurrent words, themes, or concepts, analysing the meaning and relationships between these.

## Results

### Study population

38 patients were approached for recruitment over 13 months, all of whom were found to be eligible. Two patients did not wish to enrol: one patient declined due to anxiety during the consultation (related to the diagnosis, not the study or technology proposed), and one declined recruitment without giving a reason. The 36 included patients were randomised as follows: 17 (47.2%) to standard 2D monitor with subsequent questionnaire (2D group), and 19 (52.8%) to standard 2D monitor with additional MR visualisation and subsequent questionnaire (MR group).

There were no differences in age, gender, handedness, education level, previous use of immersive technology, tumour type, and tumour location (laterality and lobe) between the two groups (Table 1).

**Table 1:** Patient demographics and comparison between 2D ground (standard 2D consultation) and MR group (additional mixed reality consultation)

	2D Group (*n* = 17)	MR Group (*n* = 19)	*P* value
Age (mean [SD])	50 (15)	54 (16)	0.515^*^
Gender			0.175^#^
Female	6 (35.3%)	11 (57.9%)	
Male	11 (64.7%)	8 (42.1%)	
Handedness			0.259^#^
Left	3 (17.6%)	1 (5.3%)	
Right	13 (76.5%)	18 (94.7%)	
Ambidextrous	1 (5.9%)	0	
Education level			0.128^#^
No qualification	0	2 (10.6%)	
Vocational qualification, e.g. BTEC/NVQ/diploma	3 (17.6%)	2 (10.5%)	
Lower secondary school, e.g. GCSE or O level	5 (29.4%)	4 (21.1%)	
Higher secondary education, e.g. GCE, A level or similar	1 (5.9%)	6 (31.6%)	
Degree level or above	8 (47.1%)	5 (26.3%)	
Previous use of immersive technology			0.830^#^
None	10 (58.8%)	13 (68.4%)	
Virtual reality	6 (35.3%)	5 (26.3%)	
Augmented reality	1 (5.9%)	1 (5.3%)	
Mixed reality	0	0	
Tumour type			0.332^#^
Meningioma	4 (23.5%)	6 (31.6%)	
Glioma WHO grade I/II	6 (35.3%)	7 (36.8%)	
Glioma WHO grade III/IV	2 (11.8%)	4 (21.1%)	
Brain metastasis	0	2 (10.5%)	
Other	5 (29.4%)	0	
Tumour location - Laterality			0.388^#^
Right	6 (35.5%)	10 (52.6%)	
Left	8 (47.1%)	8 (42.1%)	
Crossing the midline	3 (17.6%)	1 (5.3%)	
Tumour location - lobe			0.237^#^
Frontal lobe	5 (29.4%)	9 (47.4%)	
Parietal lobe	3 (17.6%)	5 (26.3%)	
Occipital lobe	0	0	
Temporal lobe	5 (29.4%)	1 (16.7%)	
Cerebellum	3 (17.6%)	1 (5.3%)	
Other	1 (5.9%)	3 (15.9%)	
Duration of visualisation of imaging sequences			
Duration in minutes of visualisation of pre-operative imaging via 2D monitor (median [IQR])	2 (2–3)	2 (2–3)	0.944^+^
Duration in minutes of visualisation of pre-operative imaging via mixed reality headset (median [IQR])	-	3 (2–4)	-
Total duration in minutes of visualisation of pre-operative imaging (median [IQR])	2 (2–3)	5 (4–6)	< 0.001^+^
Issues with equipment			0.087^#^
Yes	0	1 (5.3%)	
No	17 (100%)	18 (94.7%)	

^*^Independent t-test; ^#^Pearson Chi-Square test; ^+^Mann-Whitney U test

### Feasibility outcomes

Additional MR visualisation during outpatient neurosurgical consultations was deemed successful and feasible. Before clinic, the software automated sequence superimposition and auto-segmentation did not require any user adjustments. Recruitment rate was 94.7%, intervention fidelity was 100%, instrument completion rate was 100%, and attrition rate was 0%.

The median time spent reviewing the MRI sequences on the 2D computer monitor was 2 (IQR 2–3) minutes in the 2D Group and 2 (IQR 2–3) minutes in the MR Group (*p* = 0.944) (Table 1). After randomisation, patients in the MR Group spent an additional median of 3 (IQR 2–4) minutes reviewing the same sequences via the MR headsets. The total time spent visualising pre-operative imaging was 2 (IQR 2–3) in the 2D group and 5 (IQR 4–6) minutes in the MR group (*p* < 0.001). The mean total duration of the consultation in both groups was 30 min.

No issues or system failures were encountered with the 2D monitor visualisation in either group. However, one issue was noted in MR group: one of the MR headsets disconnected from the hospital WiFi network during the consultation. This was resolved in < 1 min by switching the device off and on, which automatically reconnected the device.

**Fig. 3:**
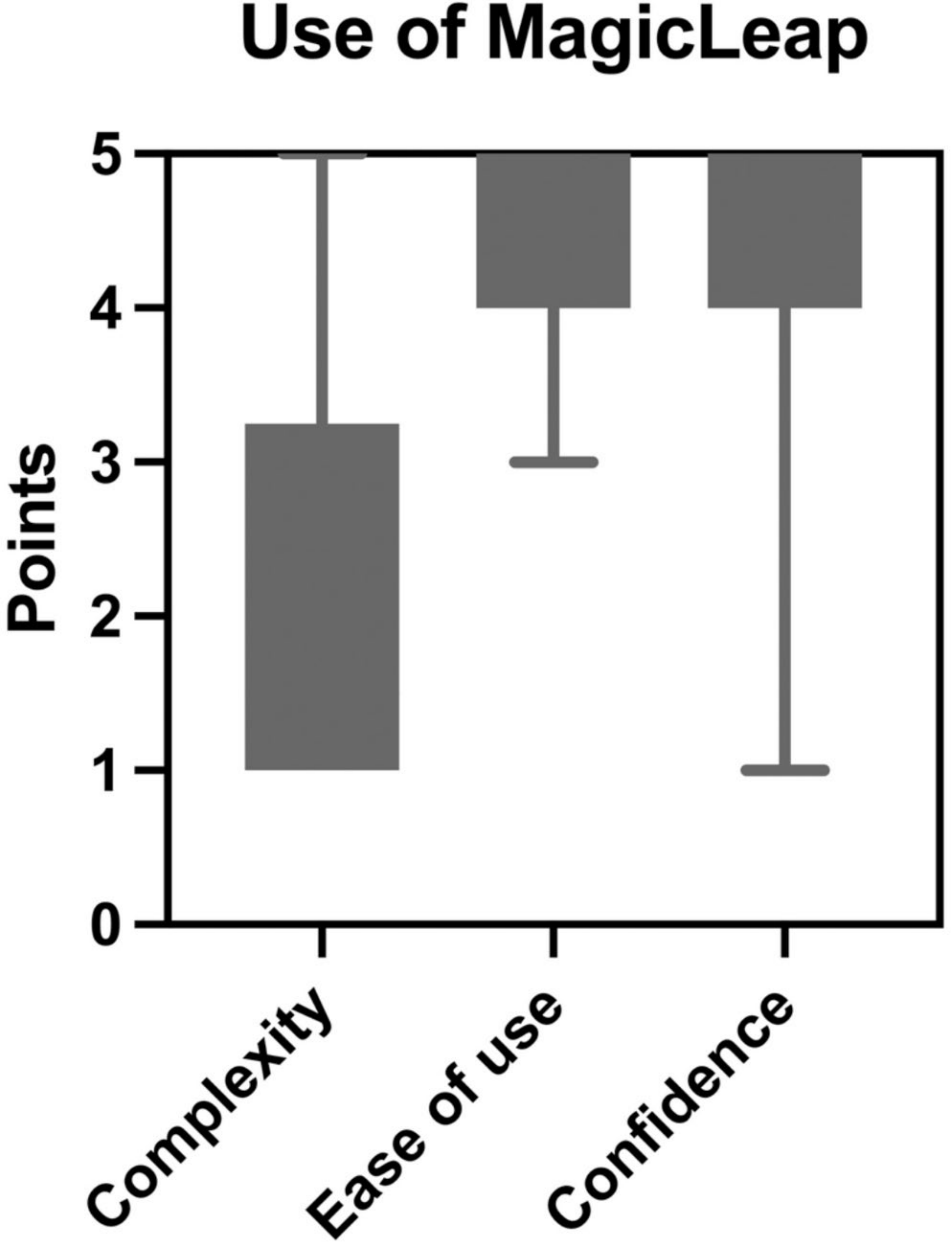
Box-plot figure with overlaid individual data points of patients in MR (2D monitor and mixed reality) self-reporting their perception of the mixed reality technology by reporting on the perceived level of complexity, ease of use and confidence in the use of the devices. The triangles represent the points awarded by each Patient for the various measured outcomes. There were no missing data points

Patients did not find the device complex (median score of 2 (IQR 1–4)), found it easy to use (median score of 5 (IQR 4–5)) and gained confidence in its use during the session (median score of 5 (IQR 4–5)) (Fig. 3).

None of the patients reported any side effects associated with simulators whilst using the MR hardware.

Table 2 reports the feasibility outcomes of methodological issues for feasibility research [23, 24].

**Table 2:** Summary of findings against 14 methodological issues for feasibility research

Methodological items	Findings	Evidence
1. What factors influenced eligibility and what proportion of those approached were eligible?	Ineligibility was not found in any of the participants approached for recruitment.	36 agreed to participate, and only two declined to consent. More may have consented if the sample size was larger.
2. Was recruitment successful?	Yes. Recruitment success was defined as 80% of eligible participants agreeing and being recruited into the study. A larger sample size is possible with increasing the number of recruiting lead clinicians and outpatient clinics.	36 out of 38 (94.7%) eligible participants agreed to take part and were recruited.
3. Did eligible participants consent?	Yes. 36 out of 38 (94.7%) eligible participants agreed to consent.	Only two did not wish to consent due to anxiety at the time of consultation (*n* = 1) and without giving a reason (*n* = 1).
4. Were participants successfully randomised?	Yes. Randomisation process worked well.	Table 1 shows no baseline group differences.
5. Were blinding procedures adequate?	No blinding was undertaken.	-
6. Did participants adhere to the intervention?	Yes. Successful adherence to the intervention was defined as at least 80% of the MR group successfully completing the consultation	All randomised participants completed the MR consultation.
7. Was the intervention acceptable to the participants?	Participants were keen to engage with the MR intervention. Acceptability was measured by refusal to engage with the MR consultation.	All randomised participants engaged with the MR consultation. Eligible participants who wished not to be recruited did not do so because of the MR consultation.
8. Was it possible to calculate intervention costs and duration?	An economic evaluation was not conducted.	-
9. Were outcome assessments completed?	Yes.	All participants completed the post-consultation questionnaire.
10. Were outcomes measured those that were the most appropriate outcomes?	All outcomes were deemed valid and appropriate.	Participants-completed forms were fully complete with no missing data points.
11. Was retention to the study good?	Successful retention in the study was defined by less than 10% attrition rate.	36 (100%) participants were successfully retained throughout the trial.
12. Were the logistics of running a multi-centre trial assessed?	No. This was a single-centre feasibility trial.	-
13. Did all components of the protocol work together?	The components of the trial and the intervention itself worked in this feasibility study.	Adherence to the intervention and study processes met the pre-determined criteria and show feasibility of progressing to full RCT, if needed.
14. Did the feasibility/pilot study allow a sample size calculation for the main trial?	No. A sample size for a future full RCT was not calculated from the data in this study.	While our study suggests trends, meaningful effect size estimates are not possible given inherent imprecision of the data at small sample sizes.

### Secondary outcomes

#### Consultation-based outcomes

**Fig. 4:**
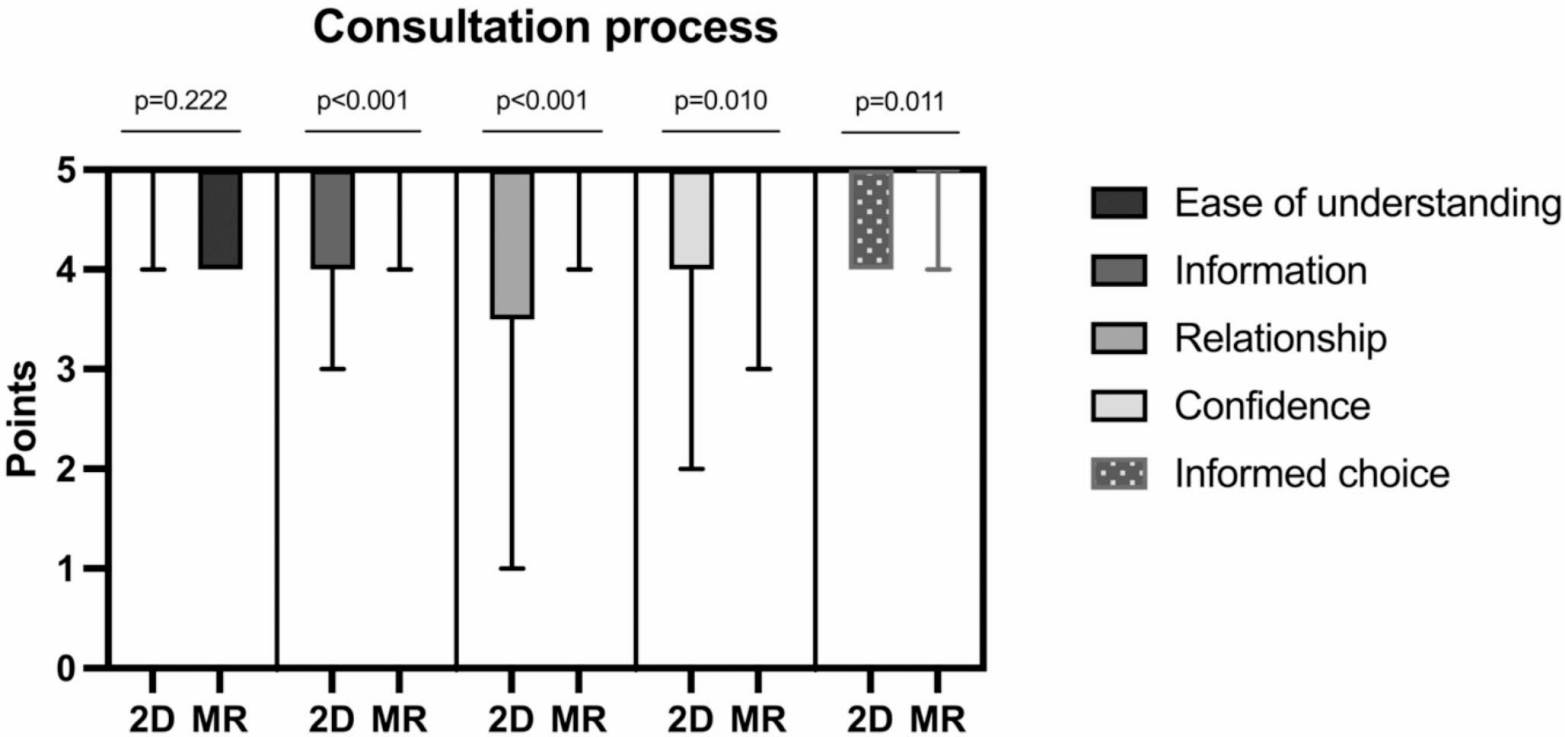
Box and whiskers graph comparing the domain of the consultation perceived by patients between the 2D group (*n* = 17, left in each column; 2D monitor) and the MR group (*n* = 19, right in each column; 2D monitor and mixed reality). The reported *p* values have been obtained via Mann-Whitney U test. This reports patients’ self-reported perception of their involvement in the treatment decision-making process, level of information received, satisfaction with their chosen treatment choice and confidence in the decision-making process

Both groups reported similar ease of understanding of the information provided during the consultation (*p* = 0.222) (Fig. 4) However, MR Group patients ranked higher satisfaction with the level of information received (median score of 5 (IQR 5–5) vs. the Standard 2D Group (5 (IQR 4–5)) (*p* < 0.001), better patient-doctor relationship (5 (IQR 5–5)) vs. 2D Group (4.5 (IQR 3.25-5)) (*p* < 0.001), greater confidence in the decision reached during the consultation (5 (IQR 5–5)) vs. 2D Group (5 (IQR 4–5)) (*p* = 0.010), and higher perception of having made an informed choice about their treatment (5 (IQR 5–5)) vs. 2D Group (5 (IQR 4–5)) (Table 3).

**Table 3:** Patient self-reported score in the administered questionnaire

Statements	2D Group					MR Group				
	*Strongly disagree*	*Disagree*	*Neutral*	*Agree*	*Strongly agree*	*Strongly disagree*	*Disagree*	*Neutral*	*Agree*	*Strongly agree*
*Satisfaction with communication* (COMRADE scale (Combined Outcome Measure for Risk communication And treatment Decision making Effectiveness))										
1. The doctor made me aware of the different treatments available.	0%	0%	0%	29.40%	70.60%	0%	0%	0%	36.90%	63.20%
2. The doctor gave me the chance to express my opinions about the different treatments available.	0%	0%	11.80%	17.60%	70.60%	0%	0%	5.30%	21.10%	73.70%
3. The doctor gave me the chance to ask for as much information as I needed about the different treatment choices.	0%	0%	5.90%	29.40%	64.70%	0%	0%	5.30%	15.80%	78.90%
4. The doctor gave me enough information about the treatment choices available.	0%	0%	17.60%	17.60%	64.70%	0%	0%	0%	15.80%	84.20%
5. The doctor gave enough explanation of the information about treatment choices.	0%	0%	11.80%	23.50%	64.70%	0%	0%	0%	15.80%	84.20%
6. The information given to me was easy to understand.*	0%	0%	0%	17.60%	82.40%	0%	0%	0%	26.30%	73.70%
7. I know the advantages of treatment or not having treatment.	0%	0%	11.80%	23.50%	64.70%	0%	0%	5.30%	21.10%	73.70%
8. I know the disadvantages of treatment or not having treatment.	0%	0%	11.80%	23.50%	64.70%	0%	0%	10.50%	26.30%	63.20%
9. The doctor gave me a chance to decide which treatment I thought was best for me.	0%	0%	17.60%	29.40%	52.90%	0%	0%	21.10%	15.80%	63.20%
10. The doctor gave me a chance to be involved in the decisions during the consultation.	0%	0%	11.80%	41.20%	47.10%	0%	0%	10.50%	21.10%	68.40%
*Confidence in decision* (COMRADE scale (Combined Outcome Measure for Risk communication And treatment Decision making Effectiveness))										
11. Overall, I am satisfied with the information I was given.*	0%	0%	5.90%	29.40%	64.70%	0%	0%	0%	10.50%	89.50%
12. My doctor and I agreed about which treatment (or no treatment) was best for me.	0%	0%	5.80%	23.50%	70.60%	0%	0%	10.50%	10.50%	78.90%
13. I can easily discuss my condition again with my doctor.*	5.90%	5.90%	11.80%	23.50%	52.90%	0%	0%	0%	15.80%	84.20%
14. I am satisfied with the way in which the decision was made in the consultation.	0%	0%	0%	29.40%	70.60%	0%	0%	0%	10.50%	89.50%
15. I am sure that the decision made was the right one for me personally.*	0%	5.90%	11.80%	23.50%	58.80%	0%	0%	5.30%	10.50%	84.20%
16. I am satisfied that I am adequately informed about the issues important to the decision.	0%	0%	0%	35.30%	64.70%	0%	0%	0%	10.50%	89.50%
17. It is clear which choice is best for me.	0%	17.60%	23.50%	11.80%	47.10%	0%	15.8%	0%	15.80%	68.40%
18. I am aware of the treatment choices I have.	0%	5.90%	11.80%	17.60%	64.70%	0%	0%	15.80%	15.80%	68.40%
19. I feel an informed choice has been made.*	0%	0%	5.90%	29.40%	64.70%	0%	0%	15.80%	10.50%	73.70%
20. The decision shows what is most important to me.	5.90%	5.90%	5.90%	29.40%	52.90%	0%	0%	10.50%	15.80%	73.70%
*Learning and engagement*										
1. I felt like this experience helped me learn about my condition.*	0%	0%	23.50%	17.60%	58.80%	0%	0%	0%	10.50%	89.50%
2. I feel confident about what my condition means for me.*	5.90%	5.90%	5.90%	23.50%	58.80%	0%	0%	0%	26.30%	66.70%
3. I enjoyed learning about my condition.	0%	5.90%	17.60%	23.50%	52.90%	5.30%	0%	5.30%	21.10%	68.40%
4. I understand what will happen next regarding my treatment for this condition.	0%	0%	11.80%	29.40%	58.80%	0%	0%	0%	15.80%	84.20%
5. I would like to learn more about my condition in the future.	0%	0%	5.90%	17.60%	76.50%	0%	5.30%	5.30%	26.30%	63.20%
*Modified System Usability Scale (m-SUS)*										
1. I think that I would like to use this ML device frequently.	-	-	-	-	-	0%	5.30%	0%	47.40%	47.40%
2. I found the ML device unnecessarily complex.*	-	-	-	-	-	47.40%	26.30%	5.30%	5.30%	15.80%
3. I thought the ML device was easy to use.*	-	-	-	-	-	0%	0%	5.30%	31.60%	63.20%
4. I think that I would need the support of a technical/clinical person to be able to use this ML device.	-	-	-	-	-	10.50%	10.50%	10.50%	31.60%	36.80%
5. I would imagine that most people would learn to use this ML device very quickly.	-	-	-	-	-	0%	0%	5.30%	42.10%	52.60%
6. I found the ML device very cumbersome to use.	-	-	-	-	-	36.80%	57.90%	5.30%	0%	0%
7. I felt very confident using the ML device.*	-	-	-	-	-	5.30%	10.50%	0%	31.60%	52.60%
8. I needed to learn a lot of things before I could get going with this VR device.	-	-	-	-	-	26.30%	36.80%	5.30%	10.50%	21.10%
*Simulator Sickness Questionnaire* [26]										
						*None*	*Slight*	*Moderate*	*Severe*	
1. General discomfort	-	-	-	-	-	100%	0%	0%	0%	-
2. Fatigue	-	-	-	-	-	100%	0%	0%	0%	-
3. Headache	-	-	-	-	-	100%	0%	0%	0%	-
4. Eye strain	-	-	-	-	-	100%	0%	0%	0%	-
5. Difficulty focusing	-	-	-	-	-	100%	0%	0%	0%	-
6. Salivation increasing	-	-	-	-	-	100%	0%	0%	0%	-
7. Sweating	-	-	-	-	-	100%	0%	0%	0%	-
8. Nausea	-	-	-	-	-	100%	0%	0%	0%	-
9. Difficulty concentrating	-	-	-	-	-	100%	0%	0%	0%	-
10. Fullness of the Head	-	-	-	-	-	100%	0%	0%	0%	-
11. Blurred vision	-	-	-	-	-	100%	0%	0%	0%	-
12. Dizziness with eyes open	-	-	-	-	-	100%	0%	0%	0%	-
13. Dizziness with eyes closed	-	-	-	-	-	100%	0%	0%	0%	-
14. *Vertigo	-	-	-	-	-	100%	0%	0%	0%	-
15. **Stomach awareness	-	-	-	-	-	100%	0%	0%	0%	-
16. Burping	-	-	-	-	-	100%	0%	0%	0%	-

* Vertigo is experienced as loss of orientation with respect to vertical upright

** Stomach awareness is usually used to indicate a feeling of discomfort which is just short of nausea

#### Educational value

**Fig. 5:**
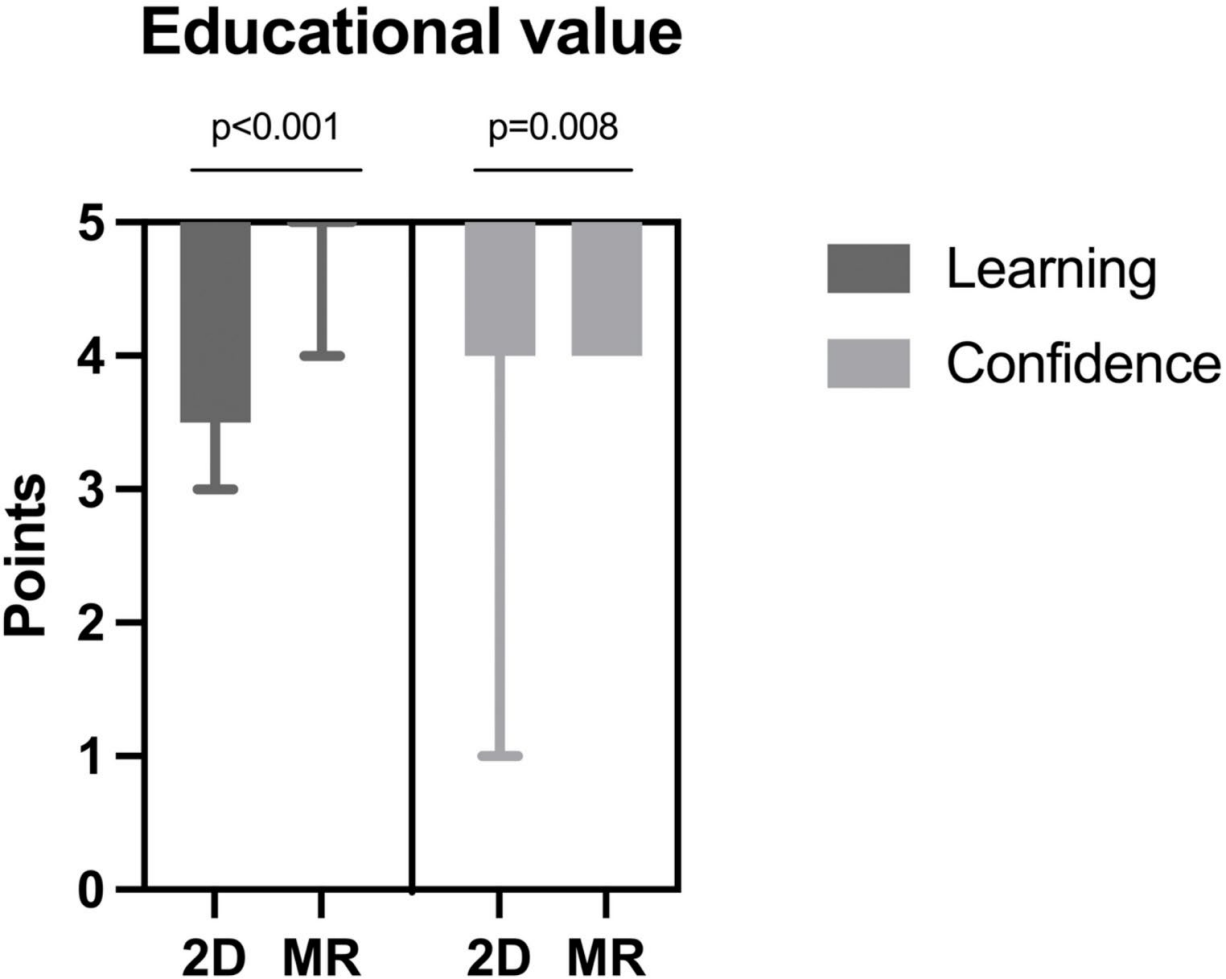
Box and whiskers graph comparing the educational value perceived by patients between the 2D group (*n* = 17, left in each column; 2D monitor) and the MR group (*n* = 19, right in each column; 2D monitor and mixed reality). This highlights the patients’ self-reported perception of the educational value and confidence in their understanding of their pathology. The reported *p* values have been obtained via Mann-Whitney U test

Patients in the MR group reported greater understanding of their disease (median score of 5 (IQR 5–5)) compared to 2D group (5 (IQR 3.25-5)) (*p* < 0.001). Furthermore, MR group patients reported higher confidence in their understanding of their condition with a median score 5 (IQR 5–5) compared to a 5 (IQR 4–5) in the 2D group (*p* = 0.008) (Fig. 5).

### Individual experiences

9 patients provided written (*n* = 3) or verbal (*n* = 6) comments on their consultation. 2D group patients focused on the difficulty of understanding the images on the monitor screen.

Patient 26 (67, male, no previous VR/MR experience): “*I’ll take your word for it. I can’t tell the difference.*”.

In contrast, MR group patients focused on the usefulness of the technology to better understand the tumour location and intended surgery.

Patient 27 (61, female, no previous VR/MR experience): “*I get it now; before it was just a light blob on the screen. This is an excellent tool for perspective.*“.

Patient 4 (54, female, no previous VR/MR experience): “*On the screen*,* it is difficult to understand where the tumour is*,* especially with the switch between right and left. Then*,* you can only see one slice and you don’t know how high up or down it is inside the brain. I would use this again.*“.

Furthermore, one patient from the MR group highlighted the usefulness of this technology in confirming she had made the right decision for her cancer treatment.

Patient 21 (52, female, previous VR gaming): “[You] *need someone to set it up. Seeing the tumour helped me make the decision* [for surgery], *and seeing how close it was to the primary cortex confirmed I have made the right decision.*”.

Of note, the main concern about the use of this technology was raised by patients wearing glasses.

Patient 4 (54, female, no previous VR/MR experience): “[It] *was slightly uncomfortable on top of my glasses and felt unstable on the nose*”.

## Discussion

Effective consultation is central to patient-centred care, bringing together the ethical, legal, and administrative healthcare delivery requirements and constructing the patient-doctor relationship [29]. Immersive technologies more intuitively display the imaging and reduce patients’ cognitive workload, which may help to improve patients’ understanding and enhance the consultation process. However, they are not employed in routine clinical practice because of the limited evidence into their efficacy and potential benefit and concerns around the feasibility of implementation and adoption.

This study has demonstrated the feasibility of additional MR visualisation of patient-specific imaging during routine outpatient neurosurgical consultation. In addition, it has shown potential benefits to patients’ experience of the consultation and information transfer. Patient benefit is two-fold: firstly, patients directly benefit from an enhanced understanding of their condition and medical literacy; secondly, patients consistently report a positively enhanced patient-doctor relationship [14, 15]. These findings will inform future studies evaluating this technology in a wider neurosurgical healthcare setting.

This seems especially beneficial in neuro-oncology where patients report the diagnosis of a brain tumour as shocking and subsequently failing to remember most of the discussed information [11, 12]. The use of MR visualisation is also a natural technological progression given that both software infrastructure and hardware adopted are routinely used in most neurosurgical departments as standard-of-care for brain tumour surgery planning and intra-operative neuronavigation. Additional benefits of the MagicLeap MR visualisation hardware that facilitate feasibility are minimal training requirements for all members of the research team, rapid image loading for each patient, and intuitive head mounting and positioning for patients.

The brevity of the additional MR visualisation (median additional duration of 3 min) would appear to be realistic to implement within a standard new patient outpatient appointment (typically 30 min in the UK). Our study purposefully aimed to deliver a pragmatic immersive technology intervention so that it would be implementable in the real-world setting of clinical practice and demonstrated likely benefit at even short usage durations. In contrast, other research groups have evaluated prolonged use of supplementary or alternative immersive and interactive technology visualisation in their intervention groups [3, 13–15]. We do not believe the prolonged duration of immersive technology interventions, from 10 min or beyond, is likely to be feasible to implement within the context of a standard clinic appointment; especially true in follow-up appointments (typically 15 min in the UK).

Our patient population randomised to MR visualisation reported high levels of ease of use and confidence in using the device, despite having received no prior training on this MR hardware and with most patients in both groups having had limited exposure to immersive technologies in their personal lives. This suggests that digital technology readiness, including digital access, usage and literacy is high among this patient population, something that is regularly cited as a concern among digital health researchers and developers [30]. In addition, our findings of higher satisfaction with the level of information received, a positive patient-doctor relationship, and greater confidence in the decision reached in the intervention group aligns with data from other cases of immersive technologies us, such as VR, which has also been shown to improve understanding in neurosurgery [14, 31].

### Limitations and next steps

Whilst these findings demonstrate early signal of efficacy with one consultant neurosurgeon at a single institution, limitations are recognised.

Firstly, randomisation occurred following surgical informed consent. This was done to minimise the impact of new technology on the conventional surgical consent process and for logistical reasons, and it is supported by similar studies [15, 32]. Whilst having a limitation in that part of the exposure happened before randomisation, as highlighted in the feasibility data, in a future definitive RCT this will be adjusted by randomising patients prior to the start of the consultation in clinic.

Secondly, blinding was not possible, and participants receiving the intervention were aware of their randomisation allocation with resultant risk for bias in the qualitative assessment of the secondary outcomes and utility of the tool. Third, it is difficult to quantify the exact contribution of immersive technologies to the self-reported improved patients’ understanding. It could be argued that patients simply benefitted from more time spent with the consultant and repetition of the information, albeit with a different visualisation medium. Nonetheless, identifying an association between MR visualisation and increased patient understanding is beyond the scope of this early feasibility RCT, which has provided a successful feasibility assessment of this technology in controlled clinical practice. The next stage of this research programme will be a multi-centre IDEAL Stage 2b RCT comparing 2D visualisation to stand-alone MR visualisation in two separate groups, which will provide further feasibility assessment and address key uncertainties for designing a definitive study.

Whilst being commonly perceived that providing in-depth information about surgical pathology and risks may augment patients’ anxiety, anxiety doesn’t increase after patients are given additional information with multimedia platforms or immersive technology [14, 33–35]. However, we did not specifically ask this in our MR patient group, and future studies should incorporate this aspect.

Studies show that disengagement and reluctance to spend more time talking about a condition or procedure might be related to a combination of cultural, behavioural, and personal factors rather than not being interested in engaging with technologies in general [14]. This is evidenced by only one patient refusing trial recruitment after having reviewed the MRI sequences on the 2D monitor: their refusal was non-specific to the MR technology but to the consultation. Future studies will include data on why some patients might not want to engage with immersive technologies.

Furthermore, our clinical implementation of this specific hardware recognised the limitation of wearing it with glasses which led to either an uncomfortable fit on patients’ face or pressure on patients’ frames. This is limitation of this specific device, especially since the interchangeable spectacle-graded inserts are expensive to purchase, and it would rely on patients remembering their glasses prescription.

Lastly, whilst we cannot draw definitive conclusions at this stage as this was an early feasibility study in a wider research program, this study will inform a larger, multi-centre randomised controlled trial. This RCT will provide a feasibility assessment of the intervention and address key uncertainties for designing a definitive study to evaluate the clinical effectiveness of MR and 3D visualisation on patients’ understanding and empowerment in shared decision-making.

## Conclusion

This study demonstrates the feasibility and possible benefits of incorporating MR visualisation into neurosurgical consultations. MR technology provides an immersive and interactive experience, enhancing patients’ understanding of their pathology and treatment options compared to traditional 2D visualisation. Undoubtedly, it is difficult to quantify the exact contribution of immersive technologies via more time spent with the consultant, repetition of the information, or with a different visualisation medium. However, the qualitative feedback provides an indication of the mechanism by which patients perceived that MR contributed to improved understanding. Ultimately, this study has provided feasibility insights for a multi-centre randomised controlled trial. This trial will provide further feasibility assessment and address key uncertainties for designing a definitive study investigating the role and impact of MR visualisation on patient empowerment and decision-making in neurosurgery.

## Electronic supplementary material

Below is the link to the electronic supplementary material.

**Supplementary Material 1**: **Video 1**: This video demonstrates the use of immersive technology, e.g. Magic Leap headsets, in the outpatient neurosurgery clinic setting. The consultant (right) is demonstrating the pathological anatomy to the simulated patient (left). The second half of the video displays the first-person viewing experience through the headsets. The simulated patient and surgeon consented to the publication of their image

## Data Availability

No datasets were generated or analysed during the current study.
